# The safety and efficacy of topical clove oil for the treatment of naturally occurring *Sarcoptes scabiei* var. *canis* infestation in dogs

**DOI:** 10.3389/fvets.2026.1899457

**Published:** 2026-08-10

**Authors:** Hongwei Hu, Lixia Dai, Yuchao Ma, Xiaoyan Yu, Xiaorong Yang, Xiaolou Miao, Jiyu Zhang, Xiaofei Shang

**Affiliations:** 1 College of Veterinary Medicine, Gansu Agricultural University, Lanzhou, China; 2 Lanzhou Institute of Husbandry and Pharmaceutical Sciences, Chinese Academy of Agricultural Sciences, Lanzhou, China; 3 Lanzhou Animal Disease Prevention and Control Center, Lanzhou, China; 4 School of Life Sciences and Food Engineering, Hebei University of Engineering, Handan, China

**Keywords:** clove oil, dog, efficacy, safety, sarcoptic mange

## Abstract

**Introduction:**

Canine sarcoptic mange is a highly contagious parasitic skin disease of dogs. On the basis of previous *in vitro* investigations confirming the acaricidal activity of clove oil, this study evaluated the safety and efficacy of topical clove oil for the treatment of dogs naturally infested with *Sarcoptes scabiei* var. *canis*.

**Methods:**

A total of 100 naturally infected dogs were randomly assigned to 7% clove oil-treated groups, a positive control group, and an untreated control group. Therapeutic efficacy was assessed using mite counts, reduction rates, and clinical scores.

**Results and discussion:**

Following a 14‑day treatment, the decline in the comprehensive infection index reached 93.25% (high dose), 85.12% (medium dose), 84.18% (positive control), and 65.23% (low dose). At D14, significant between-group differences were observed in mite-burden score (H(4) = 58.381, *P* < 0.001), mite count (H(4) = 76.161, *P* < 0.001), and MDD score (H(4) = 72.487, *P* < 0.001). Mite-free rate, complete MDD resolution rate, and cure rate also differed significantly among groups (all *P* < 0.001), with the high-dose group showing the greatest efficacy. The exploratory CII reduction rate likewise differed significantly among groups (H(4) = 79.052, *P* < 0.001). Reductions in mite count and MDD score were positively correlated (Spearman’s r_s_ = 0.727, adjusted *P* < 0.001). Notably, mite reduction was tightly linked to the alleviation of dermatitis symptoms. Hematological and biochemical abnormalities observed before treatment, including elevated white blood cell and globulin levels, gradually returned toward normal ranges following clove oil therapy. Safety pharmacology studies in rats demonstrated that topical administration of clove oil at the different doses caused no apparent adverse effects on the cardiovascular, respiratory, or central nervous systems. These results indicate that topical clove oil is a safe and effective treatment for dogs naturally infested with *S. scabiei* var. *canis* and supports its potential for further investigation.

## 1 Introduction

Sarcoptic mange is a highly contagious and intensely pruritic skin disease caused by the burrowing mite *Sarcoptes scabiei* (1, 2). This mite infests a wide range of hosts, including humans (3), dogs and cats (4), pigs (5), rabbits (6), sheep (7), and many other animals, with host specificity determined by different subspecies. Among them, *S. scabiei* var. *canis* is one of the most common ectoparasites affecting dogs worldwide. Infection typically results in severe pruritus, inflammation, excoriation, hyperkeratosis, and progressive skin lesions, and may further lead to secondary bacterial pyoderma and chronic dermatitis (8, 9).

Canine sarcoptic mange affects dogs of all breeds, ages, and sexes and is characterized by a high degree of contagion. Importantly, zoonotic transmission can occur, and humans in close contact with infested dogs may also develop pruritic skin lesions (10). Lesions most commonly appear on the periocular region, ear pinnae, elbows, and hocks, and may gradually spread to other body areas as the disease progresses. Regional epidemiological surveys show that the prevalence of canine sarcoptic mange varies drastically across different environments, ranging from below 5% in well-managed household pet populations to over 26% in unvaccinated stray and shelter dog groups; the widespread disease brings persistent costs including repeated medication, secondary infection therapy and cross-species zoonosis intervention (11, 12). In North America, sarcoptic mange is among the most frequently diagnosed dermatological diseases in veterinary teaching hospitals. Although definitive diagnosis relies on microscopic identification of mites or their eggs in skin scrapings, detection can be challenging due to the low mite burden and intermittent shedding.

Currently, several chemical acaricides are widely used for the treatment and control of canine mange in veterinary practice, including selamectin, moxidectin, and isoxazoline compounds such as fluralaner (2). These agents are administered either topically or orally and differ in their mechanisms of action and therapeutic efficacy. However, increasing concerns regarding drug resistance, adverse effects, and environmental sustainability have stimulated interest in alternative control strategies derived from natural resources. In this context, plant essential oils have attracted growing attention because of their favorable ecotoxicological properties and demonstrated antiparasitic activity (11, 12). Notably, natural acaricidal components exert antiparasitic effects through unique mechanisms and molecular targets that are distinctly different from those of conventional chemical acaricides. This discrepancy in action modes greatly reduces the probability of cross-resistance development between natural acaricides and existing chemical treatments, endowing natural product with prominent advantages for the rotation and combined application of acaricidal regimens to alleviate drug resistance.

Clove (Eugenia caryophyllata Thunb.), a member of the Myrtaceae family, is widely used as a spice and natural preservative. Its essential oil, commonly known as clove oil, contains a variety of bioactive constituents with antibacterial, antiparasitic, antifungal, and anticancer activities. Eugenol, *β*-caryophyllene, and eugenyl acetate have been identified as its principal components (13). In 2007, Fichi et al. reported that clove oil exhibited acaricidal activity both *in vivo* and *in vitro* (14). Treatment with clove oil successfully cured six rabbits infested with *Psoroptes cuniculi*, showing no significant difference compared with a commercial acaricide (Acacerulen R®), and achieved significant mite mortality at a concentration of 0.16%. Ninsanit et al. (15) demonstrated acaricidal activity of clove oil against S. scabiei. Among various plant essential oils with potential acaricidal effects, clove oil was specifically selected in this study based on our previous mechanistic research basis. Our group has systematically clarified the targeted acaricidal mechanism of eugenol, the dominant bioactive component of clove oil. We verified that eugenol exerts acaricidal action by binding to NADH dehydrogenase chain 2 and inhibiting the activity of mitochondrial respiratory chain complex I in the oxidative phosphorylation pathway, ultimately triggering energy metabolism failure and mite death. In particular, compared with the classic mitochondrial inhibitor rotenone, eugenol possesses superior biosafety and highly selective toxicity against mite cells (11). These unique mechanistic advantages and safety characteristics distinguish clove oil from other reported essential oils and provide a solid theoretical basis for its further clinical development. However, despite these promising findings, the therapeutic efficacy of clove oil in the treatment of canine sarcoptic mange remains insufficiently studied, and its safety profile, particularly following topical administration, has not been comprehensively evaluated.

In this study, we aimed to evaluate the therapeutic efficacy of different doses of clove oil in the treatment of canine sarcoptic mange and to assess its safety through safety pharmacology studies, thereby providing a scientific basis for subsequent clinical trials.

## 2 Materials and methods

### 2.1 Chemicals and ethics

Clove oil was purchased from Yuanye Bio. Co. (purity>85%, Shanghai, China, Lot: H18J7M9197), and storage conditions at 4 °C in amber vials. Before the experiment, the chemical composition of clove oil was performed using GC–MS, which contained eugenol (83.3%), next were eugenyl acetate (5.9%) and *β*-caryophyllene (4.0%). The TIC picture was provided in Supplementary Figure S1.

Amitraz solution (12.5%, Jiangsu Ouke Animal Pharmaceutical Co., Ltd., China). All procedures involving animals were reviewed and approved by the Institutional Animal Care and Use Committee (IACUC) of Lanzhou Institute of Husbandry and Pharmaceutical Sciences, Chinese Academy of Agricultural Sciences (Approval No. 2024-061, No. 2025–024).

### 2.2 The efficacy of clove oil topically for the treatment of naturally infested dogs

#### 2.2.1 Study design

The study was conducted in accordance with the Technical Guidelines for Veterinary Chemical and Traditional Chinese Medicine Research (16). Sample size was calculated using a two-sample proportion test (G*Power 3.1) based on an expected 0% cure rate in untreated controls vs. ≥80% in the high-dose eugenol group (ɑ = 0.05, power = 0.80), yielding a minimum of 18 dogs/group. Accounting for a 10% dropout rate, 20 dogs/group were enrolled (*n* = 100). Primary comparison focused on the high-dose vs. untreated control groups; other pairwise analyses were exploratory. Sensitivity analyses (FAS/PPS) will mitigate potential bias from dropouts ≤10%.

A total of 100 dogs (collected from dog breeding facilities and stray dog shelters in Lanzhou, China) meeting the inclusion criteria were enrolled and randomly assigned into five groups (A-E), with 20 dogs per group using a computer-generated random number sequence (Table 1). The randomization sequence was generated by an investigator who was not involved in animal enrollment, treatment administration, or outcome assessment. Allocation concealment was maintained using sequentially numbered, opaque, sealed envelopes, which were opened only after each dog had been confirmed eligible and enrolled. Group A served as the untreated control group and was included solely for efficacy comparison during the predefined 14-day observation period. Groups B-D received tropical administration of the different doses of clove oil, which was prepared using soybean oil. Among these three groups, group B received a low dose of clove oil, group C received a medium dose and group D received a high dose. Group E served as the positive control group treated with amitraz, which was diluted to 0.05% using water. The clove oil formulation consisted of 7% (w/v) clove oil diluted in soybean oil. Soybean oil was selected as the vehicle due to its established safety profile in veterinary topical applications, which alone served as the negative control. Detailed group allocation and treatment regimens are presented in Table 2. Following completion of the study, all dogs, including those in the untreated control group, received appropriate acaricidal treatment, achieved clinical recovery, and were returned to their original breeding facilities or shelters.

**Table 1:** Breed distribution of enrolled dogs.

Species	No. of dogs	Proportion (%)
Chinese Native Dog	68	68.0
Poodle	15	15.0
Samoyed	2	2.0
Akita	2	2.0
Labrador Retriever	1	1.0
Pembroke Welsh Corgi	5	5.0
Golden Retriever	2	2.0
French Bulldog	1	1.0
American Bully	1	1.0
Doberman Pinscher	1	1.0
German Shepherd Dog	1	1.0
Alaskan Malamute	1	1.0

**Table 2:** Experimental groups and treatment protocol.

Group	No. of dogs	Treatment protocol*
Control (A)	20	--
Low-dose Clove oil (B)	20	It was topically applied to the infected skin areas at a dosage of 0.05 mL/cm^2^
Middle-dose Clove oil (C)	20	It was topically applied to the infected skin areas at a dosage of 0.1 mL/cm^2^
High-dose Clove oil (D)	20	It was topically applied to the infected skin areas at a dosage of 0.2 mL/cm^2^
Amitraz (E)	20	It was topically applied to the infected skin areas at a dosage of 0,05% with 0.1 mL/cm^2^

*All treatments were administered once every other day with four applications.

The study duration was 14 days. To ensure animal welfare, all dogs received routine veterinary care throughout the study period. Animals showing severe clinical deterioration, secondary infections requiring immediate intervention, or other conditions compromising welfare would receive appropriate veterinary treatment and be withdrawn from the study if necessary. During the study, dogs were observed daily for general health status, including food and water intake, temperature, respiratory rate, heart rate, and behavior. All adverse events were recorded. To alleviate distress without compromising the study, non-pharmacological supportive care was provided; however, antipruritic or acaricidal agents were withheld until the end of the study. All untreated dogs received full therapeutic intervention (ivermectin) immediately after the 14-day observational period.

Prior to treatment, each dog was weighed and blood samples were collected for hematological and biochemical analyses, and the severity of mite infestation at affected skin was quantitatively assessed. The day of first administration was defined as Day 0 (D0). Treatments were administered every other day for a total of four applications, on Days 0 (D0), 2 (D2), 4 (D4), and 6 (D6). After the final administration, the study was concluded on Day 14 (D14). At study completion, body weight measurement and blood sampling were repeated. Dogs were evaluated using the mite-induced dermatitis in dogs (MDD) scoring system, which was evaluated according to MDD Scoring Criteria (Table 3). All adverse events were recorded, and safety evaluation was conducted based on treatment-related adverse reactions or clinical abnormalities observed during the experimental period (9, 16, 17).

**Table 3:** Clinical symptom score of MDD.

Clinical symptoms	0 (None)	1 (Mild)	2 (Moderate)	3 (Severe)
Pruritus Severity	No scratching/licking	Occasional, minimal behavior	Frequent, interfering with some activities	Constant and intense, severely affecting quality of life
Scaling Extent	None or Minimal	Localized (≤2 sites), sparse	Multifocal (3–4 sites), obvious patches	Generalized (≥5 sites), heavy coverage
Alopecia Extent	None	Patchy, <10% of body surface	Multifocal, 10–50% of body surface	Generalized, >50% of body surface
Erythema/Exudation	None	Localized, mild erythema	Marked erythema with mild exudation	Generalized erythema with severe exudation/ infection

#### 2.2.2 Inclusion, exclusion and withdrawal criteria

*Inclusion criteria:* (1) Presence of pruritic skin lesions, including crusts, erythema, papules, vesicles, or scaling, with mite infestation confirmed by microscopic examination of skin scrapings; (2) No history of prophylactic or therapeutic treatment for mange within the preceding 3 months; (3) Non-pregnant status; (4) Absence of severe concurrent diseases, other than mange, that could interfere with study objectives or outcome assessments; (5) No history of ovariectomy, castration, or other surgical procedures within 15 days prior to initiation of treatment.

*Exclusion criteria:* (1) Prior treatment that could potentially affect the observation or evaluation of efficacy endpoints; (2) Concurrent physiological or pathological conditions that might interfere with the assessment or interpretation of study outcomes; (3) Poor compliance, including aggressive temperament or inability to cooperate with drug administration or sample collection; (4) Planned procedures or interventions during the study period that could affect study procedures or results; (5) Presence of severe systemic diseases that were not adequately controlled.

*Withdrawal criteria:* (1) Enrollment of dogs found not to meet the inclusion criteria; (2) Premature withdrawal after receiving less than 10% of the planned treatment dose; (3) Occurrence of accidental illness during the study that could influence study outcomes; (4) Other serious protocol deviations.

#### 2.2.3 Parasitological and clinical examinations

Parasitological examinations were conducted on Days 0 and 14 using a light microscope (DM500, Leica, Germany). Mite counts and relevant clinical parameters were assessed at each time point. Skin scrapings were collected from affected areas and examined microscopically to confirm mite infestation. Detailed, three sampling sites (3 cm^2^ each) were randomly selected from the infected areas of each dog. The collected material was placed on a glass slide, mixed with mineral oil, and covered with a coverslip. Samples were examined under an optical microscope. Five fields of view were counted per sample, and the total number of live mites was recorded. Skin scrapings were initially screened under a light microscope at 40 × magnification to locate mites with precise counting. A mite was defined as viable based on the presence of active movement, turgid morphology, and intact appendages. All clinical examinations and parasitological evaluations were performed by two trained investigators who were blinded to the treatment allocation throughout data collection and analysis. Each sample was independently scored according to the criteria in Table 4 (18–20). Blood samples were obtained for hematological (Mindray BC-2800Vet, Mindray, China) and biochemical analyses (Celercare V5, MNCHIP, China).

**Table 4:** Microscopic mite burden score.

Microscopic findings (per field of view)	Score	Diagnostic criteria
No mites or eggs detected	0	Negative: No mites detected in all fields
Few observed	1	Positive: 1–2 live mites/nymphs/larvae
Moderate numbers observed	2	Positive: 3–5 live mites
Numerous observed	3	Positive: >5 live mites

#### 2.2.4 Comprehensive infection index and therapeutic efficacy evaluation

In this study, a composite infection index (CII) was preliminarily developed as an exploratory indicator integrating multiple clinical and parasitological parameters to evaluate therapeutic efficacy. Notably, this self-defined CII has not been systematically validated against authoritative standard criteria, which constitutes a limitation of the current evaluation system.

$\begin{array}{l} \mathrm{CII}=\mathrm{MDD}\;\text{the total clinical symptom score}+\text{the total mite}\;\\ \text{burden score} \end{array}$ .

$\begin{array}{l} \text{The reduction rate of}\;\mathrm{CII}\;(\%)=\\ {}[\begin{array}{l} \left(\mathrm{Pre}-\text{treatment}\;\mathrm{CII}\;\text{index}-\text{Post}-\text{treatment}\;\mathrm{CII}\;\text{index}\right)\\ /\mathrm{Pre}-\text{treatment}\;\mathrm{CII}\;\text{index} \end{array}]\times 100\% \end{array}$ .

Therapeutic efficacy criteria: Cure: Total clinical symptom score decreased to 0–1, and the total mite burden score decreased to 0. Effective: Reduction rate of the CII was ≥30%. Invalid: Reduction rate of the CII was <30%, or worsening of symptoms and microscopic findings was observed.

### 2.3 Safety pharmacology evaluation of clove oil

#### 2.3.1 Animals

Forty SD rats (purchased from Sichuan Vitals Biotech Co., Ltd., China) were randomly assigned according to body weight into four groups: low- (0.1 mL/cm^2^), medium-(0.3 mL/cm^2^), and high-dose clove oil (0.5 mL/cm^2^) groups, and a negative control group (0.1 mL/cm^2^), and there is 10 rats per group with equal numbers of males and females. The clove oil formulation consisted of 7% (w/v) clove oil diluted in soybean oil. Soybean oil was selected as the vehicle due to its established safety profile in veterinary topical applications, which alone served as the negative control. In accordance with the Technical Guidelines for Veterinary Drug Research (VDEC, 2022), three dose levels were selected for safety pharmacology evaluation, corresponding to approximately 1-, 3-, and 5-fold of the proposed clinical dose (0.1 mL/cm^2^).

In accordance with the OECD draft guideline Repeated Dose Dermal Toxicity: 21/28-day Study (No. 410) (21), the treated skin area for topical formulations should not exceed 10% of the total body surface area. Twenty-four hours before the first administration, dorsal hair was removed from the treatment area (5 cm × 6 cm), divided equally on both sides of the spine (5 cm × 3 cm per side). The test formulations or vehicle were applied to multilayer gauze and affixed to the shaved area.

#### 2.3.2 Effects on the central nervous system of clove oil

Within 4 h after the final administration, rats were closely observed for general behavior, posture, pupil response, gait, salivation, tremors, and other neurological signs using a multichannel physiological signal acquisition and processing system (BL-420F/RM6240EC, Chengdu Instrument Factory, China). Animals were further monitored daily for 7 days following the last dose. In addition, central nervous system effects were evaluated using the open-field test and the climbing pole test.

##### 2.3.2.1 Spontaneous locomotor activity of clove oil

Spontaneous activity was assessed before dosing and at 0.5 h after the final administration using an SA215 open-field test system (Suzhou Sciences Biotechnology Co., Ltd., China). Locomotor activity frequency, total distance traveled, and mean velocity were recorded over a 3-min observation period.

##### 2.3.2.2 Climbing pole test

At 0.5 h after the final administration, rats were subjected to the climbing pole test. Each rat was placed head-down on the top of a vertically fixed metal rod and allowed to descend naturally. Performance was scored as follows: Grade 0: normal stepwise descent; Grade 1: sliding down the pole; Grade 2: inability to grasp the pole; Grade 3: loss of righting reflex.

#### 2.3.3 Effects on the cardiovascular system

Heart rate was measured before dosing, on the day following the final administration, and 1 week after the final administration using a multichannel physiological signal acquisition system. Rats were anesthetized by intraperitoneal injection of tribromoethanol, and then the electrocardiograms and heart rate were recorded within 20 min after anesthesia.

#### 2.3.4 Effects on respiratory function of clove oil

The respiratory rate, rhythm, and depth were recorded using a multichannel physiological signal acquisition system.

### 2.4 Statistical analysis

Statistical analyses were performed using IBM SPSS Statistics26.0, GraphPad Prism10.4.2. *Post-hoc* body-weight-adjusted sensitivity analyses were performed in Python 3.13.5 using statsmodels 0.14.6 and SciPy 1.17.0. Before inferential analyses, continuous variables were assessed for normality using the Shapiro–Wilk test and Q-Q plots, and homogeneity of variance was evaluated using Levene’s test. Ordinal or non-normally distributed outcomes, including MDD scores, mite-burden scores, mite counts, and the exploratory CII, were summarized as medians with interquartile ranges (IQRs). Within-group comparisons between D0 and D14 were performed using the Wilcoxon signed-rank test. Between-group comparisons at D14 and comparisons of reduction rates were performed using the Kruskal-Wallis test followed by Dunn-Bonferroni adjusted pairwise comparisons. Categorical therapeutic outcomes, including mite-free status, complete MDD resolution, and cure rate, were compared among groups using the chi-square test. Pairwise comparisons were further performed using Fisher’s exact test with Bonferroni correction. Odds ratios (ORs) and 95% confidence intervals (CIs) were calculated using the Haldane-Anscombe correction when zero-cell counts were present. Mite-free status was defined as a mite-burden score of 0 with no mites detected at D14. Complete MDD resolution was defined as a D14 MDD score of 0–1. Cure was defined as both complete MDD resolution and mite-free status at D14.

To account for baseline body-weight imbalance, *post-hoc* sensitivity analyses used body-weight-adjusted models for the principal D14 efficacy outcomes. Median regression with robust standard errors analyzed D14 MDD score, mite-burden score, and exploratory CII; HC3 robust linear regression analyzed ln(D14 mite count + 1); and Firth penalized logistic regression analyzed mite-free status, complete MDD resolution, and cure at D14. Models included treatment group, baseline body weight, and, where applicable, the corresponding D0 outcome. The untreated control group served as the reference category, and treatment vs. control comparisons used Bonferroni-adjusted *p* values.

Repeated continuous variables, including hematological, biochemical, cardiovascular, respiratory, and open-field parameters, were analyzed using Gaussian repeated-measures models with an exchangeable working correlation structure. Treatment group, time, and the group × time interaction were included as model terms. *Post-hoc* within-group changes and treatment vs. control changes were adjusted using the Bonferroni method. Spearman’s rank correlation analysis was used to evaluate the association between parasitological improvement and clinical improvement. Correlation p values were adjusted using the Holm-Bonferroni method. Exact *p* values, 95% CIs, and effect sizes were reported where appropriate. Effect sizes were expressed as r for Wilcoxon signed-rank tests, Kendall’s W for Kruskal-Wallis tests, Cramer’s V for categorical outcomes, and Hedges’g for treatment vs. control changes in repeated continuous outcomes. A two-sided *p* value < 0.05 was considered statistically significant.

## 3 Results

### 3.1 The efficacy of clove oil topically for the treatment of naturally infested dogs

#### 3.1.1 General clinical observations

A total of 100 dogs were enrolled for efficacy evaluation. The cohort comprised predominantly Chinese Native Dogs (68.0%, *n* = 68), Toy Poodles (15.0%, *n* = 15), and Alaskan Malamutes (1.0%, *n* = 1), among others. The mean age was 5.30 years (range: 1–11 years), and the mean body weight was 12.91 kg (range: 2.73–47.83 kg; Table 1). Baseline body weight differed slightly among groups (Kruskal-Wallis test, H(4) = 10.062, *p* = 0.039, Kendall’s W = 0.102), but the core clinical and parasitological indicators of disease severity were comparable before treatment. In the *post-hoc* body-weight-adjusted sensitivity analyses, the overall treatment-group effect remained significant for each principal D14 efficacy outcome and for the exploratory CII (all *p <* 0.001). Baseline body weight was not statistically significant in any adjusted model. Detailed adjusted estimates are presented in Supplementary Table S5. During the study, body weight, temperature, respiratory rate, and heart rate remained within normal physiological ranges in all treatment groups. No significant differences were detected in body temperature, respiratory rate, or heart rate before and after treatment. No skin irritation was found. No dogs were withdrawn during the 14-day study period. Food intake, water consumption, general behavior, and local skin tolerance remained clinically acceptable in all treated groups. No treatment-related skin irritation or clinically relevant adverse abnormalities were observed during the study period (Table 5).

**Table 5:** Baseline characteristics and baseline disease severity of dogs.

Variable	Control	Low-dose	Middle-dose	High-dose	Amitraz	Statistic	*p* value	Effect size
Age, years	5.50 (3.00–7.00)	4.50 (3.00–7.25)	5.00 (3.00–6.00)	6.00 (3.00–7.25)	6.00 (4.00–8.00)	H(4) = 1.662	0.798	Kendall’s W = 0.017
Body weight at D0, kg	12.18 (9.57–15.93)	8.46 (4.27–11.47)	13.62 (9.13–15.58)	13.45 (9.79–17.25)	16.79 (12.23–20.18)	H(4) = 10.062	0.039	Kendall’s W = 0.102
MDD score at D0	10.00 (7.75–12.00)	10.00 (8.00–12.00)	10.00 (8.00–12.25)	11.50 (7.00–13.00)	10.50 (8.75–12.00)	H(4) = 0.461	0.977	Kendall’s W = 0.005
Mite-burden score at D0	2.00 (1.00–3.00)	2.00 (1.00–3.25)	2.50 (1.00–3.00)	2.00 (1.00–3.00)	2.00 (1.00–3.25)	H(4) = 0.128	0.998	Kendall’s W = 0.001
Mite count at D0	8.00 (5.00–10.00)	12.00 (8.00–15.00)	10.50 (8.00–15.25)	10.50 (8.00–15.25)	10.00 (8.00–14.00)	H(4) = 6.677	0.154	Kendall’s W = 0.067
CII at D0	12.50 (9.75–14.25)	12.00 (10.00–14.25)	12.00 (10.75–15.00)	14.00 (9.50–16.00)	12.50 (11.00–14.25)	H(4) = 0.223	0.994	Kendall’s W = 0.002
Sex, male/female	10/10	12/8	6/14	7/13	11/9	χ^2^(4) = 5.395	0.249	Cramer’s V = 0.232
WBC at D0	19.00 (15.25–21.92)	20.90 (17.65–28.50)	18.55 (16.02–25.57)	22.00 (19.88–25.45)	19.40 (15.75–28.20)	H(4) = 4.805	0.308	Kendall’s W = 0.049
RBC at D0	8.73 (7.82–9.40)	9.97 (6.64–11.33)	9.12 (7.97–10.09)	9.91 (8.38–11.11)	8.34 (7.28–9.64)	H(4) = 4.516	0.341	Kendall’s W = 0.046
GLO at D0	35.60 (24.95–40.03)	33.50 (30.40–43.08)	38.45 (32.70–44.23)	34.65 (30.30–48.05)	36.75 (31.90–42.00)	H(4) = 2.798	0.592	Kendall’s W = 0.028
A/G at D0	1.10 (0.90–1.60)	1.10 (0.90–1.30)	1.00 (0.80–1.10)	1.05 (0.80–1.30)	1.00 (0.88–1.23)	H(4) = 1.351	0.853	Kendall’s W = 0.014
Temperature at D0	37.05 (35.98–37.65)	36.60 (36.27–37.90)	37.05 (36.58–37.82)	36.80 (35.95–38.02)	37.25 (36.08–37.92)	H(4) = 0.280	0.991	Kendall’s W = 0.003
Heart rate at D0	126.50 (103.25–134.25)	122.50 (105.25–128.00)	107.00 (97.75–123.50)	110.00 (98.00–124.75)	121.00 (109.75–124.50)	H(4) = 5.254	0.262	Kendall’s W = 0.053
Respiratory rate at D0	24.00 (20.00–26.25)	22.50 (19.00–26.25)	26.00 (19.00–27.25)	24.50 (18.00–27.00)	21.00 (19.75–23.50)	H(4) = 1.484	0.829	Kendall’s W = 0.015

Data are presented as median (interquartile range) unless otherwise indicated. Group comparisons were performed using the Kruskal-Wallis test, except sex distribution, which was analyzed using the chi-square test. Effect sizes are Kendall’s W or Cramer’s V.

#### 3.1.2 Effect of clove oil on mite counts in dogs with sarcoptic mange

Mite-burden scores were significantly reduced from D0 to D14 in all clove oil-treated groups and in the amitraz group according to Wilcoxon signed-rank tests, whereas the untreated control group showed no meaningful parasitological improvement (Supplementary Table S1). At D14, mite-burden scores differed significantly among groups (Kruskal-Wallis test, H(4) = 58.381, *p < 0.001*, Kendall’s W = 0.590; Supplementary Table S2). Dunn-Bonferroni *post-hoc* analysis showed that all treatment groups had significantly lower D14 mite-burden scores than the untreated control group (Supplementary Table S3). Actual mite counts showed a similar pattern. At D14, mite counts differed significantly among groups (Kruskal-Wallis test, H(4) = 76.161, *p < 0.001*, Kendall’s W = 0.769). Compared with the untreated control group, all clove oil-treated groups and the amitraz group had significantly lower mite counts after Dunn-Bonferroni adjustment. No significant difference in D14 mite count was detected between the high-dose clove oil group and the amitraz group.

The mite-free rate at D14 also differed significantly among groups (χ^2^(4) = 52.922, *p* < 0.001, Cramer’s V = 0.727). By Day 14, 2, 12, and 20 dogs in the low-, medium-, and high-dose clove oil groups, respectively, were mite-free, compared with 10 dogs in the positive control group (Tables 6, 7). This result indicated that clove oil treatment markedly reduced mite counts in dogs with sarcoptic mange, with efficacy comparable to that of amitraz after 14 days of treatment.

**Table 6:** Main efficacy outcomes at D14 and reduction rates.

Outcome	Control	Low-dose	Middle-dose	High-dose	Amitraz	Statistic	*p* value	Effect size
MDD score at D14	10.00 (8.00–12.25)	3.50 (2.00–5.25)	1.00 (1.00–2.00)	1.00 (1.00–1.00)	1.00 (1.00–2.00)	H(4) = 72.487	<0.001	Kendall’s W = 0.732
Mite-burden score at D14	2.00 (2.00–3.25)	0.50 (0.00–1.00)	0.00 (0.00–0.25)	0.00 (0.00–0.00)	0.00 (0.00–1.00)	H(4) = 58.381	<0.001	Kendall’s W = 0.590
Mite count at D14	7.00 (6.00–9.25)	1.00 (0.75–2.00)	0.00 (0.00–0.00)	0.00 (0.00–0.00)	0.00 (0.00–0.00)	H(4) = 76.161	<0.001	Kendall’s W = 0.769
CII at D14	13.00 (10.75–15.25)	4.00 (2.75–6.00)	2.00 (1.00–2.00)	1.00 (1.00–1.00)	2.00 (1.00–3.00)	H(4) = 76.688	<0.001	Kendall’s W = 0.775
MDD reduction rate (%)	−4.17 (−9.32–0.00)	67.53 (43.33–83.33)	85.16 (80.00–88.89)	91.67 (89.72–92.45)	85.16 (82.95–89.17)	H(4) = 73.275	<0.001	Kendall’s W = 0.740
Mite-burden score reduction rate (%)	0.00 (−100.00–0.00)	90.00 (66.67–100.00)	100.00 (93.75–100.00)	100.00 (100.00–100.00)	100.00 (75.00–100.00)	H(4) = 64.855	<0.001	Kendall’s W = 0.655
Mite-count reduction rate (%)	13.57 (−20.77–26.25)	88.19 (82.50–95.31)	100.00 (100.00–100.00)	100.00 (100.00–100.00)	100.00 (100.00–100.00)	H(4) = 77.676	<0.001	Kendall’s W = 0.785
CII reduction rate (%)	−8.57 (−15.38–0.00)	69.91 (54.17–80.00)	85.71 (81.68–87.85)	93.54 (90.68–93.75)	84.52 (80.58–88.89)	H(4) = 79.052	<0.001	Kendall’s W = 0.799

Values are median (interquartile range). Overall comparisons were performed using the Kruskal-Wallis test. *Post-hoc* pairwise comparisons were adjusted using the Dunn-Bonferroni method. Full pairwise comparisons are provided in Supplementary Table S3.

**Table 7:** Categorical therapeutic outcomes at D14.

Outcome	Control	Low-dose	Middle-dose	High-dose	Amitraz	Statistic	*p* value	Effect size	Selected Fisher comparisons
Mite-free at D14	0/20 (0.0)	2/20 (10.0)	12/20 (60.0)	20/20 (100.0)	10/20 (50.0)	χ^2^(4) = 52.922	<0.001	Cramer’s V = 0.727	High-dose vs. Control: OR = 1681.00 (31.81–88846.11), P_adj = <0.001; High-dose vs. Amitraz: OR = 41.00 (2.18–770.12), P_adj = <0.001
Complete MDD resolution at D14	0/20 (0.0)	3/20 (15.0)	12/20 (60.0)	20/20 (100.0)	11/20 (55.0)	χ^2^(4) = 50.483	<0.001	Cramer’s V = 0.711	High-dose vs. Control: OR = 1681.00 (31.81–88846.11), P_adj = <0.001; High-dose vs. Amitraz: OR = 33.87 (1.80–636.91), P_adj = 0.002
Cure at D14	0/20 (0.0)	1/20 (5.0)	6/20 (30.0)	20/20 (100.0)	5/20 (25.0)	χ^2^(4) = 59.099	<0.001	Cramer’s V = 0.769	High-dose vs. Control: OR = 1681.00 (31.81–88846.11), P_adj = <0.001; High-dose vs. Amitraz: OR = 115.55 (5.93–2250.69), P_adj = <0.001

Data are n/total (%). Overall comparisons were analyzed using the chi-square test. Selected pairwise comparisons were performed using Fisher’s exact test with Bonferroni correction. ORs and 95% CIs were calculated using the Haldane-Anscombe correction when zero-cell counts were present.

#### 3.1.3 Effect of clove oil on mite-induced dermatitis in dogs (MDD)

**Figure 1:**
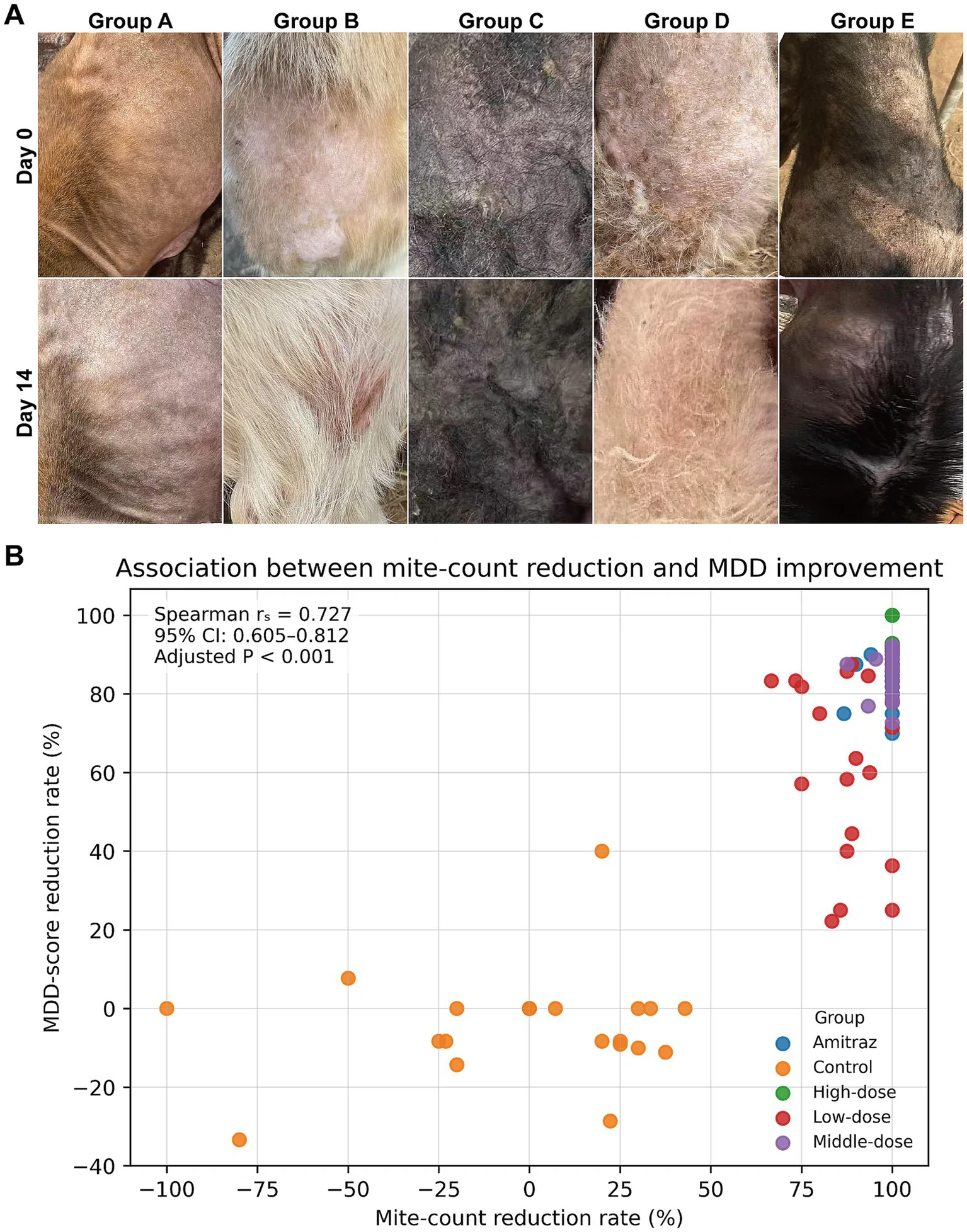
The picture of before and after treatment of dogs with sarcoptic mange **(A)** and Spearman_MiteCount_vs_MDD **(B)**.

As shown in Table 8, before treatment (Day 0), mean MDD scores were comparable among all groups, ranging from 9.80 to 10.20, with no dogs free of MDD in any group. After 14 days of treatment, marked improvements in MDD scores were observed in the clove oil-treated groups. MDD scores decreased to 3.65, 1.55, and 0.85 in the low-, medium-, and high-dose clove oil groups, respectively, compared with 10.15 in the untreated control group. Amitraz group showed a similarly low score (1.60). By D14, complete MDD resolution was observed in 0, 3, 12, 20, and 11 dogs in the control, low-dose, medium-dose, high-dose, and amitraz groups, respectively (Tables 6, 7; Figure 1). This result indicated that clove oil treatment alleviated mite-induced dermatitis in dogs, with near-complete resolution observed in the medium- and high-dose groups after 14 days.

**Table 8:** Repeated-measures model analysis of hematological, biochemical, and clinical safety parameters in dogs.

Variable	Group effect	Time effect	Group × time interaction	Key *post-hoc* result
WBC	χ^2^(4) = 8.223, *p* = 0.084	χ^2^(1) = 0.086, *p* = 0.769	χ^2^(4) = 9.133, *p* = 0.058	High-dose vs. Control: (D14-D0) difference: estimate = −6.700 (−11.088–-2.312), P_adj = 0.011, Hedges g = −0.904
RBC	χ^2^(4) = 3.805, *p* = 0.433	χ^2^(1) = 0.061, *p* = 0.805	χ^2^(4) = 1.258, *p* = 0.868	No significant treatment-vs-control change after Bonferroni correction
GLO	χ^2^(4) = 4.032, *p* = 0.402	χ^2^(1) = 1.748, *p* = 0.186	χ^2^(4) = 7.404, *p* = 0.116	No significant treatment-vs-control change after Bonferroni correction
AG	χ^2^(4) = 2.648, *p* = 0.618	χ^2^(1) = 0.270, *p* = 0.603	χ^2^(4) = 3.205, *p* = 0.524	No significant treatment-vs-control change after Bonferroni correction
Temp	χ^2^(4) = 0.323, *p* = 0.988	χ^2^(1) = 0.469, *p* = 0.494	χ^2^(4) = 3.060, *p* = 0.548	No significant treatment-vs-control change after Bonferroni correction
HR	χ^2^(4) = 4.634, *p* = 0.327	χ^2^(1) = 1.649, *p* = 0.199	χ^2^(4) = 4.309, *p* = 0.366	No significant treatment-vs-control change after Bonferroni correction
RR	χ^2^(4) = 1.880, *p* = 0.758	χ^2^(1) = 2.581, *p* = 0.108	χ^2^(4) = 4.657, *p* = 0.324	No significant treatment-vs-control change after Bonferroni correction

Repeated continuous variables were analyzed using Gaussian repeated-measures models with an exchangeable working correlation structure. Treatment group, time, and group × time interaction were included as model terms. *Post-hoc* treatment vs. control changes were adjusted using the Bonferroni method.

#### 3.1.4 Effect of clove oil on the CII

CII was analyzed as an exploratory composite index integrating clinical and parasitological parameters. CII values were significantly reduced from D0 to D14 in all clove oil-treated groups and in the amitraz group. Following the 14-day treatment, the CII in the low-, medium-, and high-dose clove oil groups were 4.20, 1.85, and 0.85, corresponding to average reduction rates of 65.23, 85.12, and 93.25%, respectively. The amitraz group demonstrated a comparable efficacy, achieving a mite burden score of 0.00 and a reduction rate of 84.18%. The results showed that treatment in the low-dose clove oil group was effective, that in the medium-dose group and the positive drug group was markedly effective, and that in the high-dose group achieved cure. At D14, CII differed significantly among groups (Kruskal-Wallis test, H(4) = 76.688, *p <* 0.001, Kendall’s W = 0.775; Table X). The CII reduction rate also differed significantly among groups (Kruskal-Wallis test, H(4) = 79.052, *p <* 0.001, Kendall’s W = 0.799; Tables 6, 7). These findings indicate that clove oil not only significantly reduces mite counts but also exerts a potent therapeutic effect on *Sarcoptes scabiei-*induced dermatitis in dogs.

#### 3.1.5 The safety of clove oil on naturally *S. scabiei* var. *canis* infestation in dogs

No dogs were withdrawn during the experimental period. Based on observations of body temperature, heart rate, and respiratory rate, no significant differences were detected between pre- and post-treatment values in any group. Before treatment, the white blood cell (WBC) counts were 19.34 in the control group, 22.04 in the low-dose group, 21.95 in the medium-dose group, 21.97 in the high-dose group, and 22.00 in the positive control group. After treatment, WBC counts decreased in all treated groups, whereas no obvious change was observed in the control group. Following treatment the RBC counts increased in all treated groups, while no clear increase was observed in the control group. Serum globulin (GLO) levels and the albumin-to-globulin ratio (A/G) showed improvement after treatment (Table 8). More importantly, no clinically relevant adverse reactions were observed in dogs treated with either clove oil or amitraz throughout the treatment period. Graphical summaries of the hematological and serum biochemical profiles at the two prespecified sampling time points are presented in Supplementary Figures S2, S3. These findings indicate that the tested dogs tolerated the clove oil, and that it demonstrated a favorable safety profile.

### 3.2 Safety evaluation of clove oil

#### 3.2.1 Effects of clove oil on the central nervous system in rats

Throughout the study, no abnormalities were observed in the appearance, behavior, posture, gait, or pupil response in rats treated with low, medium, or high doses of clove oil liniment. No signs of salivation, muscle tremors, or other neurological abnormalities were detected in any treatment group.

In the open-field test, repeated-measures analysis showed a significant time effect for spontaneous activity (Wald χ^2^(1) = 9.353, *p* = 0.002) and a significant group × time interaction (Wald χ^2^(3) = 8.314, *p* = 0.040; Table 9). Bonferroni-adjusted *post-hoc* comparisons showed that the high-dose group differed from the negative control group in the change in spontaneous activity (adjusted *p* = 0.030). However, no significant group × time interactions were detected for total distance traveled or average speed. In the climbing pole test, pole scores did not differ significantly among groups (Kruskal-Wallis test, H(3) = 2.053, *p* = 0.562, Kendall’s W = 0.053). Only one rat in the negative control group and one rat in the low-dose group showed Grade 1 sliding, whereas no Grade 2 or Grade 3 responses were observed. Overall, these findings did not indicate consistent treatment-related central nervous system toxicity under the present experimental conditions. Taken together, findings from general clinical observations, the climbing pole and the open-field tests indicate that topical application of clove oil did not produce detectable adverse effects on the central nervous system of rats (Table 5).

**Table 9:** Safety pharmacology outcomes in rats.

Endpoint	Effect / test term	Statistic	df	*p* value	Effect size	Interpretation
Spontaneous activity	Group	Wald χ^2^ = 1.523	3	0.677	0.019	No overall group effect
Spontaneous activity	Time	Wald χ^2^ = 9.353	1	**0.002**	0.105	Significant time effect
Spontaneous activity	Group × Time	Wald χ^2^ = 8.314	3	**0.040**	0.094	High-dose change vs. control change: adjusted *p* = 0.030
Total distance traveled	Group	Wald χ^2^ = 3.373	3	0.338	0.040	No overall group effect
Total distance traveled	Time	Wald χ^2^ = 2.837	1	0.092	0.034	No significant time effect
Total distance traveled	Group × Time	Wald χ^2^ = 3.548	3	0.315	0.042	No significant interaction
Average speed	Group	Wald χ^2^ = 3.367	3	0.338	0.040	No overall group effect
Average speed	Time	Wald χ^2^ = 2.820	1	0.093	0.034	No significant time effect
Average speed	Group × Time	Wald χ^2^ = 3.571	3	0.312	0.043	No significant interaction
Climbing pole score	Group	H = 2.053	3	0.562	0.053	No significant group difference
Heart rate	Group	Wald χ^2^ = 6.868	3	0.076	0.054	No overall group effect
Heart rate	Time	Wald χ^2^ = 0.106	2	0.949	0.001	No significant time effect
Heart rate	Group × Time	Wald χ^2^ = 2.381	6	0.881	0.019	No significant interaction
Respiratory rate	Group	Wald χ^2^ = 0.328	3	0.955	0.003	No overall group effect
Respiratory rate	Time	Wald χ^2^ = 0.840	2	0.657	0.007	No significant time effect
Respiratory rate	Group × Time	Wald χ^2^ = 6.494	6	0.370	0.051	No significant interaction

Repeated continuous endpoints were analyzed using Gaussian repeated-measures models with an exchangeable working correlation structure. Group, time, and group × time terms were included in each model. The climbing pole score was analyzed using the Kruskal-Wallis test. Effect size for repeated-measures model terms is reported as χ^2^/(χ^2^ + n); effect size for the climbing pole score is reported as Kendall’s W. *p* values <0.05 are shown in bold. The significant group × time interaction for spontaneous activity should be interpreted together with the *post-hoc* comparison; no consistent treatment-related central nervous system, cardiovascular, or respiratory toxicity was detected under the present experimental conditions.

#### 3.2.2 The effects of clove oil on heart rate in rats

The effects of clove oil on heart rate in rats are shown in Table 6. No significant differences in heart rate were observed within any treatment group when comparing values measured before dosing, on the day following the administration, and 1 week after the administration (Table 9). Heart rate values in all groups remained within the normal physiological range for rats (370–580 beats/min). These results indicate that topical application of clove oil liniment at the tested dose levels did not produce detectable effects on heart rate in rats.

#### 3.2.3 Effects of clove oil on respiratory rate in rats

The effects of clove oil on respiratory rate in rats are presented in Table 6. No marked changes in respiratory rate were observed in the low-, medium-, or high-dose clove oil groups or in the negative control group before dosing, on the day following the final administration, or 1 week after the final administration. Comparisons between each treatment group and the control group at the same time points revealed no significant differences. In addition, no statistically differences in respiratory rate were detected within the same group across different time points (*p > 0.05*). Repeated-measures analysis showed no significant group × time interaction for respiratory rate (Wald χ^2^(6) = 6.494, *p* = 0.370). No significant within-group changes remained after Bonferroni correction (Table 9). These findings indicate a favorable safety pharmacology profile of clove oil following dermal exposure, supporting its further clinical development.

## 4 Discussion

Sarcoptic mange is a contagious parasitic skin disease of dogs caused by *Sarcoptes scabiei* var. *canis*, which can lead to severe pruritus, alopecia, skin lesions, and even death if left untreated (22). Owing to the limitations of conventional acaricides, increasing attention has been directed toward plant-derived compounds as alternative control measures. Previous studies have demonstrated that several medicinal plants, including *Adonis coerulea*, *Azadirachta indica*, and *Eupatorium adenphorum*, possess acaricidal activity (23–25), suggesting their potential application in the management of sarcoptic mange. Our previous studies also showed that essential oils from *Rhododendron nivale* and oregano exhibited remarkable acaricidal activity (26, 27). Similarly, clove oil and its active compound eugenol have been reported to possess acaricidal effects (11, 14, 28). Based on these findings, the present study systematically evaluated the therapeutic efficacy and safety of clove oil in the treatment of canine sarcoptic mange, thereby supporting its potential for further clinical development.

The results demonstrated that clove oil exhibited significant therapeutic efficacy against canine sarcoptic mange. Treatment with different doses of clove oil resulted in a marked reduction in mite infestation, with the high-dose group achieved a mean mite burden score of 0.00, followed by the medium-dose group (0.30), the positive control group (0.40), and the low-dose group (0.55). The comparable efficacy observed between the medium-dose group and the positive control suggests that clove oil may serve as a promising plant-derived alternative to conventional acaricides.

From an economic perspective, representative oral isoxazoline products (e.g., sarolaner, fluralaner) are prescription-only chewable tablets, with a full therapeutic course costing approximately 70–240 RMB for medium-sized dogs; though they provide long-lasting mite control with fewer administrations, continuous treatment imposes higher financial pressure on owners. In contrast, the topical 7% clove oil relies on low-cost bulk plant oil raw materials, and the total material expense for a 14-day treatment cycle is markedly lower. Nevertheless, clove oil requires repeated manual skin application and mandatory glove use for operators, which adds extra time and labor costs. Overall, clove oil exhibits prominent raw material cost advantages for grassroots breeding and low-income pet owners, whereas isoxazolines are more suitable for families prioritizing convenient, low-frequency medication despite higher drug expenditure.

The reduction in mite burden was closely associated with the alleviation of mite density-related dermatitis (MDD), indicating that effective parasite elimination plays a key role in clinical recovery (Supplementary Table S4; Figure 1). Improvements in dermatitis severity further support the ability of clove oil to mitigate inflammatory skin lesions caused by *S. scabiei* infestation. These findings highlight the dual benefits of clove oil in controlling both parasitic load and inflammatory manifestations of the disease. Isoxazoline-based therapies represent the contemporary standard of care for canine sarcoptic mange. For instance, Carithers et al. (29) reported that a single oral administration of afoxolaner (NexGard^®^, Merial) at a 2.5 mg/kg dose achieved >98% efficacy against induced *O. cynotisn* infestations. Similarly, Beugnet et al. (30) demonstrated that oral afoxolaner treatment resulted in significantly lower mite counts compared to untreated controls at Days 28 and 56 (*p <* 0.001), with no mites recovered from treated dogs at these time points (100% efficacy). Furthermore, dogs receiving afoxolaner exhibited significantly improved lesion resolution by Day 56 relative to Day 0 (*p <* 0.05). Consistent with these findings, Romero et al. (17) showed that a single dose of fluralaner effectively eradicated scabies mites within 14 days and significantly alleviated clinical signs associated with sarcoptic mange within 21 days. Collectively, these studies confirm that isoxazoline-based therapies offer rapid parasiticidal effects at relatively low doses. However, while highly effective against mites, the resolution of cutaneous lesions with isoxazolines appears comparatively slower than that observed with clove oil. Unlike isoxazolines primarily target parasitic nervous systems, clove oil possesses inherent tissue-repair property (31).

Hematological and biochemical analyses further revealed that clove oil treatment contributed to the normalization of immune- and inflammation-related indicators. Elevated white blood cell counts and globulin levels observed prior to treatment, likely reflecting immune activation and chronic inflammatory responses induced by mite infestation and secondary infections, gradually declined following therapy. Red blood cell counts and albumin/globulin ratios also tended to return toward normal ranges, suggesting recovery from parasite-induced physiological disturbances. These trends are consistent with previous studies reporting hematological and biochemical alterations in dogs affected by sarcoptic mange (32–34).

The therapeutic effects of clove oil observed in this study may be attributed to its bioactive components, particularly eugenol, which has been reported to possess acaricidal, antimicrobial, and anti-inflammatory properties (35). Combining RNA-Seq and SPR analysis, and RNA interference assay, our group found that eugenol has been shown to inhibit complex I activity of the mitochondrial respiratory chain in the oxidative phosphorylation pathway by binding to NADH dehydrogenase chain 2, ultimately leading to mite death. In contrast to the known inhibitor rotenone, eugenol had better safety, and insect cells were particularly sensitive to eugenol (11). In addition, its anti-inflammatory activity may help reduce skin irritation, promote restoration of the skin barrier, and limit protein loss and secondary bacterial infections (36). The combined direct and indirect actions of clove oil likely account for the observed improvement in clinical signs and physiological parameters. More importantly, beside mites, clove oil and eugenol also presented the activity anti-ticks, such as *Hyalomma scupense* and *Rhipicephalus annulatus* (37, 38).

In addition to therapeutic efficacy, drug safety is a critical prerequisite for clinical application, especially for topical agents intended for repeated use. Therefore, safety pharmacology studies were conducted to evaluate the potential effects of transdermal administration of clove oil on the cardiovascular, respiratory, and central nervous systems. The results demonstrated that topical application of clove oil at different doses caused no apparent adverse effects on the cardiovascular, respiratory, or central nervous systems in rats, indicating a favorable safety profile.

## 5 Limitation of the study

The CII was used in this study as an exploratory composite measure to integrate both clinical and parasitological changes during treatment. This approach allowed simultaneous consideration of mite-induced dermatitis severity and microscopic mite burden, thereby providing a supportive overview of therapeutic response. However, CII has not been externally validated in independent canine sarcoptic mange studies, and the equal weighting of MDD score and mite-burden score may not fully reflect their relative biological or clinical importance. Therefore, CII should be interpreted as a supportive exploratory endpoint rather than as a validated primary efficacy endpoint. In the present study, treatment efficacy was primarily interpreted based on separately reported mite-burden scores, actual mite counts, MDD scores, mite-free rates, complete MDD resolution rates, and cure rates. Residual baseline variability related to body size, breed, affected skin area, or other unmeasured clinical characteristics cannot be fully excluded in this field study.Although no overt clinical adverse effects were observed following topical application of clove oil in this 14-day study, we urge caution in interpreting these findings as definitive evidence of safety. The current evaluation was limited to a short-term observational period, and the rat safety pharmacology data presented herein cannot fully replace comprehensive target-animal safety evaluations in dogs. Notably, potential issues such as chronic toxicity, delayed hypersensitivity, and systemic absorption remain unaddressed. Future investigations should prioritize extended-duration safety studies in the target species, standardized cutaneous irritation and sensitization assays, and pharmacokinetic profiling to evaluate systemic exposure. Until such data are available, the clinical use of clove oil should be considered preliminary and monitored accordingly.

A limitation of this study is the reliance on morphological identification without molecular confirmation. Due to the low parasite burden in clinical samples, sufficient biological material for DNA extraction and PCR analysis could not be obtained. While the morphological characteristics and the strict host specificity of *S. scabiei* var. Canissupport our diagnosis, we acknowledge that molecular techniques would provide a higher degree of taxonomic certainty. Future epidemiological investigations should integrate molecular tools to validate morphological findings. A critical limitation of this study is the relatively short observation window, which did not allow for the assessment of long-term outcomes, including parasite recurrence or relapse rates post-treatment. Although the short-term data are promising, they primarily serve to establish proof-of-concept rather than validate clinical utility. Therefore, these results should be viewed as supporting the need for further controlled clinical development, specifically emphasizing the necessity of extended follow-up studies to confirm durable efficacy and safety profiles before any consideration of practical clinical application.

## 6 Conclusion

Overall, these findings indicate that clove oil is effective in reducing mite infestation and alleviating clinical symptoms in dogs with sarcoptic mange. Among the three tested concentrations, topical administration of 7% clove oil at a dose of 0.1 mL/cm^2^ exhibited the most reliable therapeutic effect, which can serve as a preferential dosage reference for clinical application. In addition, considering the potential irritant properties of clove oil, personal protective equipment such as gloves is strongly recommended for animal owners during topical application to avoid unnecessary skin exposure. The favorable efficacy and safety profile observed in this study supports its potential uses and provides a scientific basis for subsequent clinical trials.

## Data Availability

The original contributions presented in the study are included in the article/Supplementary material, further inquiries can be directed to the corresponding authors.

## References

[1] Ainslie KEC Hooiveld M Wallinga J. Estimation of the epidemiological characteristics of scabies. Nat Commun. (2025) 16:10524. doi: 10.1038/s41467-025-65544-y, 41298361 PMC12658204

[2] Krieger K Heine J Dumont P Hellmann K. Efficacy and safety of imidacloprid 10% plus moxidectin 2.5% spot-on in the treatment of sarcoptic mange and otoacariosis in dogs: results of a European field study. Parasitol Res. (2005) 97:S81–8. doi: 10.1007/s00436-005-1449-9, 16228280

[3] Hengge UR Currie BJ Jager G Lupi O Schwartz RA. Scabies: a ubiquitous neglected skin disease. Lancet Infect Dis. (2006) 6:769–79. doi: 10.1016/S1473-3099(06)70654-5, 17123897

[4] Malik R McKellar Stewart K Sousa CA Krockenberger MB Pope S Ihrke P . Crusted scabies (sarcoptic mange) in four cats due to *Sarcoptes scabiei* infestation. J Feline Med Surg. (2006) 8:327–39. doi: 10.1016/j.jfms.2006.05.005, 16950639 PMC10822234

[5] Goyena E Ruiz de Ybanez R Martinez-Carrasco C Balseiro A Alonso de Vega F Casais R . On the aggregated nature of chronic *Sarcoptes scabiei* infection in adult pigs. Vet Parasitol. (2013) 192:301–6. doi: 10.1016/j.vetpar.2012.10.007, 23131577

[6] Millan J Casais R Delibes-Mateos M Calvete C Rouco C Castro F . Widespread exposure to *Sarcoptes scabiei* in wild European rabbits (*Oryctolagus cuniculus*) in Spain. Vet Parasitol. (2012) 183:323–9. doi: 10.1016/j.vetpar.2011.07.046, 21852039

[7] Rahbari S Nabian S Bahonar AR. Some observations on sheep sarcoptic mange in Tehran province, Iran. Trop Anim Health Prod. (2009) 41:397–401. doi: 10.1007/s11250-008-9203-9, 18626781

[8] Becskei C De Bock F Illambas J Cherni JA Fourie JJ Lane M . Efficacy and safety of a novel oral isoxazoline, sarolaner (Simparica™), for the treatment of sarcoptic mange in dogs. Vet Parasitol. (2016) 222:56–61. doi: 10.1016/j.vetpar.2016.02.017, 26928658

[9] Chiummo R Petersen I Plehn C Zschiesche E Roepke R Thomas E. Efficacy of orally and topically administered fluralaner (Bravecto®) for treatment of client-owned dogs with sarcoptic mange under field conditions. Parasit Vectors. (2020) 13:524. doi: 10.1186/s13071-020-04395-6, 33069261 PMC7568370

[10] Russell RC Otranto D Wall RL. The Encyclopedia of Medical & Veterinary Entomology. Wallingford: CABI (2013).

[11] Shang XF Dai LX Yang CJ Guo X Liu YQ Miao XL . A value-added application of eugenol as acaricidal agent: the mechanism of action and the safety evaluation. J Adv Res. (2020) 34:149–58. doi: 10.1016/j.jare.2020.12.010, 35024187 PMC8655235

[12] Shang XF Dai LX Cao XY Ma YD Gulnaz I Miao XL . Natural products in antiparasitic drug discovery: advances, opportunities and challenges. Nat Prod Rep. (2025) 42:1419–58. doi: 10.1039/d5np00007f, 40501439

[13] David K Anumudu CK Onyeaka H. Antibiofilm effect of nisin and clove oil (*Syzygium aromaticum*) on *Escherichia coli* biofilms. Heliyon. (2026) 12:e44501. doi: 10.1016/j.heliyon.2026.e44501

[14] Fichi G Flamini G Giovanelli F Otranto D Perrucci S. Efficacy of an essential oil of *Eugenia caryophyllata* against *Psoroptes cuniculi*. Exp Parasitol. (2007) 115:168–72. doi: 10.1016/j.exppara.2006.07.005, 16973163

[15] Mahakittikun V Soonthornchareonnon N Foongladda S Boitano JJ Wangapai T Ninsanit P. A preliminary study of the acaricidal activity of clove oil, *Eugenia caryophyllus*. Asian Pac J Allergy Immunol. (2014) 32:46–52. doi: 10.12932/AP0342.32.1.2014, 24641290

[16] Veterinary Drug Evaluation Center (VDEC), Ministry of Agriculture and Rural Affairs of the People’s Republic of China. Technical Guidelines for Research on Veterinary Chemical Drugs and Traditional Chinese Medicines (2022 Edition). Beijing: China Agricultural Science and Technology Press (2022).

[17] Beugnet F de Vos C Liebenberg J Halos L Larsen D Fourie J. Efficacy of afoxolaner in a clinical field study in dogs naturally infested with *Sarcoptes scabiei*. Parasite. (2016) 23:26. doi: 10.1051/parasite/2016026, 27317462 PMC4912682

[18] Fourie LJ Kok DJ Du Plessis A Rugg D. Efficacy of a novel formulation of metaflumizone plus amitraz for the treatment of demodectic mange in dogs. Vet Parasitol. (2007) 150:268–74. doi: 10.1016/j.vetpar.2007.08.047, 17923331

[19] Lebon W Beccati M Bourdeau P Brement T Bruet V Cekiera A . Efficacy of two formulations of afoxolaner (NexGard® and NexGard spectra®) for the treatment of generalised demodicosis in dogs, in veterinary dermatology referral centers in Europe. Parasit Vectors. (2018) 11:506. doi: 10.1186/s13071-018-3083-2, 30201031 PMC6131853

[20] Mueller RS Bensignor E Ferrer L Holm B Lemarie S Paradis M . Treatment of demodicosis in dogs: 2011 clinical practice guidelines. Vet Dermatol. (2012) 23:86–96. doi: 10.1111/j.1365-3164.2011.01026.x, 22329600

[21] OECD ilibrary, OECD Guildelines for the testing of chemicals, section 4[EB/OL]. (2026) doi: 10.1787/20745788

[22] Nwufoh OC Sadiq NA Fagbohun O Adebiyi A Adeshina R Emmanuel E . Molecular detection and characterization of *Sarcoptes scabiei* var *canis* using skin scrapings and skin biopsies. J Parasit Dis. (2020) 45:258–62. doi: 10.1007/s12639-020-01304-7, 33746412 PMC7921259

[23] Du YH Jia RY Yin ZQ Pu ZH Chen J Yang F . Acaricidal activity of extracts of neem (*Azadirachta indica*) oil against the larvae of the rabbit mite *Sarcoptes scabiei* var. *cuniculi* in vitro. Vet Parasitol. (2008) 157:144–8. doi: 10.1016/j.vetpar.2008.07.011, 18752898

[24] Dai LX Miao XL Li B Zhang JY Pan H Shang XF. The active compounds and AChE inhibitor of the methanol extract of *Adonis coerulea* maxim against *Psoroptes cuniculi*. Vet Parasitol. (2020) 286:109247. doi: 10.1016/j.vetpar.2020.109247 32987229

[25] Nong X Ren YJ Wang JH Fang CL Xie Y Yang DY . Clinical efficacy of botanical extracts from *Eupatorium adenphorum* against the scab mites, *Psoroptes cuniculi*. Vet Parasitol. (2013) 192:247–52. doi: 10.1016/j.vetpar.2012.10.005 23107339

[26] Guo X Shang XF Li B Zhou XZ Wen H Zhang JY. Acaricidal activities of the essential oil from *Rhododendron nivale* Hook. f. and its main compund, δ-cadinene against *Psoroptes cuniculi*. Vet Parasitol. (2017) 236:51–4. doi: 10.1016/j.vetpar.2017.01.028, 28288764

[27] Shang XF Wang Y Zhou XZ Guo X Dong SW Wang DS . Acaricidal activity of oregano oil and its major component, carvacrol, thymol and p-cymene against *Psoroptes cuniculi* in vitro and in vivo. Vet Parasitol. (2016) 226:93–6. doi: 10.1016/j.vetpar.2016.07.001, 27514892

[28] Ma WR Fan YP Liu ZY Hao YJ Mou Y Liu YQ . The acaricidal activity and mechanism of eugenol on *Psoroptes cuniculi*. Vet Parasitol. (2019) 266:56–62. doi: 10.1016/j.vetpar.2018.12.012, 30736947

[29] Hameed M Rasul A Waqas MK Saadullah M Aslam N Abbas G . Formulation and evaluation of a clove oil-encapsulated nanofiber formulation for effective wound-healing. Molecules. (2021) 26:2491. doi: 10.3390/molecules26092491, 33923335 PMC8123120

[30] Carithers D Crawford J de Vos C Lotriet A Fourie J. Assessment of afoxolaner efficacy against Otodectes cynotis infestations of dogs. Parasit Vectors. (2016) 9:635. doi: 10.1186/s13071-016-1924-4, 27938395 PMC5148825

[31] Romero C Heredia R Pineda J Serrano JA Mendoza GD Trápala P . Efficacy of fluralaner in 17 dogs with sarcoptic mange. Vet Dermatol. (2016) 27:353–e88. doi: 10.1111/vde.12363, 27511592

[32] Vovk MV Huralska SV. Clinical signs and hematological changes in dogs with ectoparasitic dermatitis. Sci Mess LNU Vet Med Biotechnol. (2025) 27:17–22. doi: 10.32718/nvlvet11803

[33] Beigh SA Soodan JS Bhat AM. Sarcoptic mange in dogs: its effect on liver, oxidative stress, trace minerals and vitamins. Vet Parasitol. (2016) 227:30–4. doi: 10.1016/j.vetpar.2016.07.013, 27523934

[34] Lee SJ Lee AN Shin EB. Sarcoptic mange in reintroduced red foxes (*Vulpes vulpes*) in South Korea: case histories, clinical assessments, treatments, and pathological findings. Animals. (2025) 15:1491. doi: 10.3390/ani15101491, 40427367 PMC12108189

[35] Ulanowska M Olas B. Biological properties and prospects for the application of eugenol-a review. Int J Mol Sci. (2021) 22:3671. doi: 10.3390/ijms22073671, 33916044 PMC8036490

[36] Maurya AK Agarwal K Gupta AC Saxena A Nooreen Z Tandon S . Synthesis of eugenol derivatives and its anti-inflammatory activity against skin inflammation. Nat Prod Res. (2020) 34:251–60. doi: 10.1080/14786419.2018.1528585, 30580605

[37] Liu J Yin S Wang Z Qin J Wang D Chen H . Screening of essential oils with acaricidal activity against the poultry red mite (*Dermanyssus gallinae*) and analysis of active components. Vet Parasitol. (2025) 339:110589. doi: 10.1016/j.vetpar.2022.109712 40896850

[38] Alimia D Hajria A Jalloulib S Sebaia H. Toxicity, repellency, and anti-cholinesterase activities of bioactive molecules from clove buds *Syzygium aromaticum* L. as an ecological alternative in the search for control *Hyalomma scupense* (Acari: Ixodidae). Heliyon. (2023) 9:e18899. doi: 10.1016/j.heliyon.2023.e18899 37600394 PMC10432207

