# Supplementary material for: The safety and efficacy of topical clove oil for the treatment of naturally occurring Sarcoptes scabiei var. canis infestation in dogs
Source: Front Vet Sci. 2026 Aug 10;13:1899457. doi: 10.3389/fvets.2026.1899457 (PMC13500287; doi:10.3389/fvets.2026.1899457)
Supplement: Supplementary file 1 [file Table_1.DOCX]

**Supplementary Table S1. Normality, homogeneity of variance, and statistical method selection**

| **Dataset** | **Variable** | **Data type** | **Minimum Shapiro-Wilk P across groups** | **Groups with Shapiro P<0.05** | **Levene P** | **Final method used** |
| --- | --- | --- | --- | --- | --- | --- |
| Dogs efficacy | MDD_D14 | Ordinal score | <0.001 | 4 | NA | Kruskal-Wallis + Dunn-Bonferroni |
| Dogs efficacy | MiteScore_D14 | Ordinal score | <0.001 | 4 | NA | Kruskal-Wallis + Dunn-Bonferroni |
| Dogs efficacy | MiteCount_D14 | Count/non-normal | <0.001 | 3 | NA | Kruskal-Wallis + Dunn-Bonferroni |
| Dogs efficacy | CII_D14 | Exploratory composite score | <0.001 | 3 | NA | Kruskal-Wallis + Dunn-Bonferroni |
| Dogs efficacy | MDD_Reduction_% | Reduction rate | 0.002 | 4 | NA | Kruskal-Wallis + Dunn-Bonferroni |
| Dogs efficacy | MiteScore_Reduction_% | Reduction rate | <0.001 | 4 | NA | Kruskal-Wallis + Dunn-Bonferroni |
| Dogs efficacy | MiteCount_Reduction_% | Reduction rate | <0.001 | 3 | NA | Kruskal-Wallis + Dunn-Bonferroni |
| Dogs efficacy | CII_Reduction_% | Reduction rate | 0.013 | 1 | NA | Kruskal-Wallis + Dunn-Bonferroni |
| Dogs continuous repeated measures | WBC | Continuous repeated measure | <0.001 | 3 | 0.597 | Gaussian repeated-measures model (GEE) |
| Dogs continuous repeated measures | RBC | Continuous repeated measure | 0.127 | 0 | 0.576 | Gaussian repeated-measures model (GEE) |
| Dogs continuous repeated measures | GLO | Continuous repeated measure | 0.108 | 0 | 0.354 | Gaussian repeated-measures model (GEE) |
| Dogs continuous repeated measures | AG | Continuous repeated measure | 0.002 | 2 | 0.695 | Gaussian repeated-measures model (GEE) |
| Dogs continuous repeated measures | Temp | Continuous repeated measure | 0.040 | 1 | 0.508 | Gaussian repeated-measures model (GEE) |
| Dogs continuous repeated measures | HR | Continuous repeated measure | 0.017 | 1 | 0.564 | Gaussian repeated-measures model (GEE) |
| Dogs continuous repeated measures | RR | Continuous repeated measure | 0.133 | 0 | 0.548 | Gaussian repeated-measures model (GEE) |

Normality was assessed using the Shapiro-Wilk test within each group. Homogeneity of variance was assessed using Levene's test for continuous change variables. NA, not applicable for ordinal/count outcomes.

**Supplementary Table S2. Within-group D0-D14 comparisons using the Wilcoxon signed-rank test**

| **Outcome** | **Group** | **n** | **D0 median (IQR)** | **D14 median (IQR)** | **Median change (95% CI)** | **Wilcoxon W** | **Approx. Z** | **P value** | **Effect size r** |
| --- | --- | --- | --- | --- | --- | --- | --- | --- | --- |
| MDD score | Control | 20 | 10.00 (7.75–12.00) | 10.00 (8.00–12.25) | 0.50 (0.00–1.00) | 17.00 | -1.959 | 0.078 | 0.565 |
| MDD score | Low-dose | 20 | 10.00 (8.00–12.00) | 3.50 (2.00–5.25) | -7.00 (-8.00–-4.00) | 0.00 | -3.963 | <0.001 | 0.886 |
| MDD score | Middle-dose | 20 | 10.00 (8.00–12.25) | 1.00 (1.00–2.00) | -8.00 (-10.00–-7.00) | 0.00 | -3.946 | <0.001 | 0.882 |
| MDD score | High-dose | 20 | 11.50 (7.00–13.00) | 1.00 (1.00–1.00) | -10.50 (-11.50–-6.00) | 0.00 | -3.976 | <0.001 | 0.889 |
| MDD score | Amitraz | 20 | 10.50 (8.75–12.00) | 1.00 (1.00–2.00) | -9.00 (-10.00–-7.50) | 0.00 | -3.973 | <0.001 | 0.888 |
| Mite-burden score | Control | 20 | 2.00 (1.00–3.00) | 2.00 (2.00–3.25) | 0.00 (0.00–1.00) | 4.50 | -2.665 | 0.035 | 0.888 |
| Mite-burden score | Low-dose | 20 | 2.00 (1.00–3.25) | 0.50 (0.00–1.00) | -1.50 (-2.00–-1.00) | 0.00 | -3.970 | <0.001 | 0.911 |
| Mite-burden score | Middle-dose | 20 | 2.50 (1.00–3.00) | 0.00 (0.00–0.25) | -2.00 (-3.00–-1.00) | 0.00 | -4.104 | <0.001 | 0.918 |
| Mite-burden score | High-dose | 20 | 2.00 (1.00–3.00) | 0.00 (0.00–0.00) | -2.00 (-3.00–-1.00) | 0.00 | -4.041 | <0.001 | 0.904 |
| Mite-burden score | Amitraz | 20 | 2.00 (1.00–3.25) | 0.00 (0.00–1.00) | -2.00 (-2.50–-1.00) | 0.00 | -4.063 | <0.001 | 0.908 |
| Mite count | Control | 20 | 8.00 (5.00–10.00) | 7.00 (6.00–9.25) | -1.50 (-2.00–1.00) | 66.50 | -0.853 | 0.403 | 0.201 |
| Mite count | Low-dose | 20 | 12.00 (8.00–15.00) | 1.00 (0.75–2.00) | -10.00 (-12.00–-8.00) | 0.00 | -3.940 | <0.001 | 0.881 |
| Mite count | Middle-dose | 20 | 10.50 (8.00–15.25) | 0.00 (0.00–0.00) | -10.50 (-14.00–-8.00) | 0.00 | -3.940 | <0.001 | 0.881 |
| Mite count | High-dose | 20 | 10.50 (8.00–15.25) | 0.00 (0.00–0.00) | -10.50 (-15.00–-8.00) | 0.00 | -3.940 | <0.001 | 0.881 |
| Mite count | Amitraz | 20 | 10.00 (8.00–14.00) | 0.00 (0.00–0.00) | -9.50 (-13.50–-8.50) | 0.00 | -3.944 | <0.001 | 0.882 |
| CII | Control | 20 | 12.50 (9.75–14.25) | 13.00 (10.75–15.25) | 1.00 (0.00–2.00) | 22.00 | -2.300 | 0.027 | 0.594 |
| CII | Low-dose | 20 | 12.00 (10.00–14.25) | 4.00 (2.75–6.00) | -8.00 (-9.50–-7.00) | 0.00 | -3.955 | <0.001 | 0.884 |
| CII | Middle-dose | 20 | 12.00 (10.75–15.00) | 2.00 (1.00–2.00) | -10.00 (-12.00–-9.50) | 0.00 | -3.951 | <0.001 | 0.883 |
| CII | High-dose | 20 | 14.00 (9.50–16.00) | 1.00 (1.00–1.00) | -13.00 (-15.00–-9.00) | 0.00 | -3.971 | <0.001 | 0.888 |
| CII | Amitraz | 20 | 12.50 (11.00–14.25) | 2.00 (1.00–3.00) | -10.50 (-12.00–-9.50) | 0.00 | -3.955 | <0.001 | 0.884 |

Data are presented as median (interquartile range). Median change is presented with bootstrap 95% CI. Effect size r was calculated from the approximate Z statistic.

**Supplementary Table S3. Full Dunn-Bonferroni pairwise comparisons for efficacy outcomes**

| **Outcome** | **Comparison** | **Mean rank difference** | **Z** | **Raw P** | **Adjusted P (Bonferroni)** |
| --- | --- | --- | --- | --- | --- |
| MDD score at D14 | Control vs Low-dose | 27.925 | 3.181 | 0.001 | 0.015 |
| MDD score at D14 | Control vs Middle-dose | 51.500 | 5.867 | <0.001 | <0.001 |
| MDD score at D14 | Control vs High-dose | 68.550 | 7.809 | <0.001 | <0.001 |
| MDD score at D14 | Control vs Amitraz | 50.025 | 5.699 | <0.001 | <0.001 |
| MDD score at D14 | Low-dose vs Middle-dose | 23.575 | 2.686 | 0.007 | 0.072 |
| MDD score at D14 | Low-dose vs High-dose | 40.625 | 4.628 | <0.001 | <0.001 |
| MDD score at D14 | Low-dose vs Amitraz | 22.100 | 2.518 | 0.012 | 0.118 |
| MDD score at D14 | Middle-dose vs High-dose | 17.050 | 1.942 | 0.052 | 0.521 |
| MDD score at D14 | Middle-dose vs Amitraz | -1.475 | -0.168 | 0.867 | 1.000 |
| MDD score at D14 | High-dose vs Amitraz | -18.525 | -2.110 | 0.035 | 0.348 |
| Mite-burden score at D14 | Control vs Low-dose | 36.900 | 4.520 | <0.001 | <0.001 |
| Mite-burden score at D14 | Control vs Middle-dose | 47.025 | 5.761 | <0.001 | <0.001 |
| Mite-burden score at D14 | Control vs High-dose | 57.975 | 7.102 | <0.001 | <0.001 |
| Mite-burden score at D14 | Control vs Amitraz | 42.975 | 5.265 | <0.001 | <0.001 |
| Mite-burden score at D14 | Low-dose vs Middle-dose | 10.125 | 1.240 | 0.215 | 1.000 |
| Mite-burden score at D14 | Low-dose vs High-dose | 21.075 | 2.582 | 0.010 | 0.098 |
| Mite-burden score at D14 | Low-dose vs Amitraz | 6.075 | 0.744 | 0.457 | 1.000 |
| Mite-burden score at D14 | Middle-dose vs High-dose | 10.950 | 1.341 | 0.180 | 1.000 |
| Mite-burden score at D14 | Middle-dose vs Amitraz | -4.050 | -0.496 | 0.620 | 1.000 |
| Mite-burden score at D14 | High-dose vs Amitraz | -15.000 | -1.838 | 0.066 | 0.661 |
| Mite count at D14 | Control vs Low-dose | 29.500 | 3.612 | <0.001 | 0.003 |
| Mite count at D14 | Control vs Middle-dose | 54.500 | 6.673 | <0.001 | <0.001 |
| Mite count at D14 | Control vs High-dose | 60.250 | 7.376 | <0.001 | <0.001 |
| Mite count at D14 | Control vs Amitraz | 54.500 | 6.673 | <0.001 | <0.001 |
| Mite count at D14 | Low-dose vs Middle-dose | 25.000 | 3.061 | 0.002 | 0.022 |
| Mite count at D14 | Low-dose vs High-dose | 30.750 | 3.765 | <0.001 | 0.002 |
| Mite count at D14 | Low-dose vs Amitraz | 25.000 | 3.061 | 0.002 | 0.022 |
| Mite count at D14 | Middle-dose vs High-dose | 5.750 | 0.704 | 0.481 | 1.000 |
| Mite count at D14 | Middle-dose vs Amitraz | 0.000 | 0.000 | 1.000 | 1.000 |
| Mite count at D14 | High-dose vs Amitraz | -5.750 | -0.704 | 0.481 | 1.000 |
| CII at D14 | Control vs Low-dose | 26.500 | 2.955 | 0.003 | 0.031 |
| CII at D14 | Control vs Middle-dose | 51.575 | 5.750 | <0.001 | <0.001 |
| CII at D14 | Control vs High-dose | 73.125 | 8.153 | <0.001 | <0.001 |
| CII at D14 | Control vs Amitraz | 48.425 | 5.399 | <0.001 | <0.001 |
| CII at D14 | Low-dose vs Middle-dose | 25.075 | 2.796 | 0.005 | 0.052 |
| CII at D14 | Low-dose vs High-dose | 46.625 | 5.198 | <0.001 | <0.001 |
| CII at D14 | Low-dose vs Amitraz | 21.925 | 2.445 | 0.015 | 0.145 |
| CII at D14 | Middle-dose vs High-dose | 21.550 | 2.403 | 0.016 | 0.163 |
| CII at D14 | Middle-dose vs Amitraz | -3.150 | -0.351 | 0.725 | 1.000 |
| CII at D14 | High-dose vs Amitraz | -24.700 | -2.754 | 0.006 | 0.059 |
| MDD reduction rate (%) | Control vs Low-dose | -27.150 | -2.963 | 0.003 | 0.031 |
| MDD reduction rate (%) | Control vs Middle-dose | -48.450 | -5.287 | <0.001 | <0.001 |
| MDD reduction rate (%) | Control vs High-dose | -73.975 | -8.072 | <0.001 | <0.001 |
| MDD reduction rate (%) | Control vs Amitraz | -49.300 | -5.380 | <0.001 | <0.001 |
| MDD reduction rate (%) | Low-dose vs Middle-dose | -21.300 | -2.324 | 0.020 | 0.201 |
| MDD reduction rate (%) | Low-dose vs High-dose | -46.825 | -5.109 | <0.001 | <0.001 |
| MDD reduction rate (%) | Low-dose vs Amitraz | -22.150 | -2.417 | 0.016 | 0.157 |
| MDD reduction rate (%) | Middle-dose vs High-dose | -25.525 | -2.785 | 0.005 | 0.053 |
| MDD reduction rate (%) | Middle-dose vs Amitraz | -0.850 | -0.093 | 0.926 | 1.000 |
| MDD reduction rate (%) | High-dose vs Amitraz | 24.675 | 2.692 | 0.007 | 0.071 |
| Mite-burden score reduction rate (%) | Control vs Low-dose | -40.250 | -4.898 | <0.001 | <0.001 |
| Mite-burden score reduction rate (%) | Control vs Middle-dose | -51.000 | -6.206 | <0.001 | <0.001 |
| Mite-burden score reduction rate (%) | Control vs High-dose | -60.675 | -7.384 | <0.001 | <0.001 |
| Mite-burden score reduction rate (%) | Control vs Amitraz | -46.450 | -5.653 | <0.001 | <0.001 |
| Mite-burden score reduction rate (%) | Low-dose vs Middle-dose | -10.750 | -1.308 | 0.191 | 1.000 |
| Mite-burden score reduction rate (%) | Low-dose vs High-dose | -20.425 | -2.486 | 0.013 | 0.129 |
| Mite-burden score reduction rate (%) | Low-dose vs Amitraz | -6.200 | -0.755 | 0.451 | 1.000 |
| Mite-burden score reduction rate (%) | Middle-dose vs High-dose | -9.675 | -1.177 | 0.239 | 1.000 |
| Mite-burden score reduction rate (%) | Middle-dose vs Amitraz | 4.550 | 0.554 | 0.580 | 1.000 |
| Mite-burden score reduction rate (%) | High-dose vs Amitraz | 14.225 | 1.731 | 0.083 | 0.834 |
| Mite-count reduction rate (%) | Control vs Low-dose | -29.175 | -3.568 | <0.001 | 0.004 |
| Mite-count reduction rate (%) | Control vs Middle-dose | -55.300 | -6.762 | <0.001 | <0.001 |
| Mite-count reduction rate (%) | Control vs High-dose | -60.500 | -7.398 | <0.001 | <0.001 |
| Mite-count reduction rate (%) | Control vs Amitraz | -55.025 | -6.729 | <0.001 | <0.001 |
| Mite-count reduction rate (%) | Low-dose vs Middle-dose | -26.125 | -3.195 | 0.001 | 0.014 |
| Mite-count reduction rate (%) | Low-dose vs High-dose | -31.325 | -3.831 | <0.001 | 0.001 |
| Mite-count reduction rate (%) | Low-dose vs Amitraz | -25.850 | -3.161 | 0.002 | 0.016 |
| Mite-count reduction rate (%) | Middle-dose vs High-dose | -5.200 | -0.636 | 0.525 | 1.000 |
| Mite-count reduction rate (%) | Middle-dose vs Amitraz | 0.275 | 0.034 | 0.973 | 1.000 |
| Mite-count reduction rate (%) | High-dose vs Amitraz | 5.475 | 0.670 | 0.503 | 1.000 |
| CII reduction rate (%) | Control vs Low-dose | -25.425 | -2.773 | 0.006 | 0.055 |
| CII reduction rate (%) | Control vs Middle-dose | -50.125 | -5.468 | <0.001 | <0.001 |
| CII reduction rate (%) | Control vs High-dose | -76.700 | -8.367 | <0.001 | <0.001 |
| CII reduction rate (%) | Control vs Amitraz | -47.750 | -5.209 | <0.001 | <0.001 |
| CII reduction rate (%) | Low-dose vs Middle-dose | -24.700 | -2.694 | 0.007 | 0.071 |
| CII reduction rate (%) | Low-dose vs High-dose | -51.275 | -5.593 | <0.001 | <0.001 |
| CII reduction rate (%) | Low-dose vs Amitraz | -22.325 | -2.435 | 0.015 | 0.149 |
| CII reduction rate (%) | Middle-dose vs High-dose | -26.575 | -2.899 | 0.004 | 0.037 |
| CII reduction rate (%) | Middle-dose vs Amitraz | 2.375 | 0.259 | 0.796 | 1.000 |
| CII reduction rate (%) | High-dose vs Amitraz | 28.950 | 3.158 | 0.002 | 0.016 |

Between-group pairwise comparisons were performed using Dunn's test after the Kruskal-Wallis test. P values were adjusted using Bonferroni correction.

**Supplementary Table S4. Spearman correlation analysis between parasitological and clinical improvement**

| **Analysis** | **Variable 1** | **Variable 2** | **n** | **Spearman r_s** | **95% CI** | **Raw P** | **Adjusted P (Holm-Bonferroni)** | **Interpretation** |
| --- | --- | --- | --- | --- | --- | --- | --- | --- |
| Mite-burden score reduction vs MDD reduction | MiteScore_Reduction_% | MDD_Reduction_% | 100 | 0.595 | 0.425–0.727 | <0.001 | <0.001 | Positive correlation |
| Mite-count reduction vs MDD reduction | MiteCount_Reduction_% | MDD_Reduction_% | 100 | 0.727 | 0.605–0.812 | <0.001 | <0.001 | Positive correlation |
| D14 mite-burden score vs D14 MDD score | MiteScore_D14 | MDD_D14 | 100 | 0.586 | 0.418–0.721 | <0.001 | <0.001 | Positive correlation |
| CII reduction vs MDD reduction (supportive; CII includes MDD) | CII_Reduction_% | MDD_Reduction_% | 100 | 0.942 | 0.891–0.972 | <0.001 | <0.001 | Positive correlation |

95% CIs for Spearman correlations were estimated by bootstrap resampling. Correlation P values were adjusted using the Holm-Bonferroni method. CII correlations are supportive because CII includes the MDD score by definition.

| **Supplementary Table S5. Overall body-weight-adjusted sensitivity analyses** | | | | | | | |
| --- | --- | --- | --- | --- | --- | --- | --- |
| **Outcome** | **Model** | **Covariates** | **Global treatment effect** | **Global P value** | **Baseline-weight effect (95% CI)** | **Weight P value** | **Interpretation** |
| D14 MDD score | Median regression (τ=0.50) | Baseline body weight + corresponding D0 outcome | χ²(4)=165.576 | <0.001 | 0.000 (-0.037 to 0.037) per kg | 1.000 | Overall treatment effect remained significant; body weight was not independently associated |
| D14 mite-burden score | Median regression (τ=0.50) | Baseline body weight + corresponding D0 outcome | χ²(4)=47.815 | <0.001 | 0.000 (-0.017 to 0.017) per kg | 1.000 | Overall treatment effect remained significant; body weight was not independently associated |
| Exploratory D14 CII | Median regression (τ=0.50) | Baseline body weight + corresponding D0 outcome | χ²(4)=252.603 | <0.001 | 0.009 (-0.029 to 0.048) per kg | 0.644 | Overall treatment effect remained significant; body weight was not independently associated |
| D14 mite count | Linear regression of ln(count+1) with HC3 robust SE | Baseline body weight + ln(D0 count+1) | χ²(4)=849.936 | <0.001 | 0.998 (0.989 to 1.008) per kg | 0.746 | Overall treatment effect remained significant; body weight was not independently associated |
| Mite-free status at D14 | Firth penalized logistic regression | Baseline body weight | χ²(4)=65.262 | <0.001 | 1.016 (0.945 to 1.092) per kg | 0.667 | Overall treatment effect remained significant; body weight was not independently associated |
| Complete MDD resolution at D14 | Firth penalized logistic regression | Baseline body weight | χ²(4)=62.407 | <0.001 | 1.028 (0.961 to 1.099) per kg | 0.421 | Overall treatment effect remained significant; body weight was not independently associated |
| Cure at D14 | Firth penalized logistic regression | Baseline body weight | χ²(4)=65.738 | <0.001 | 1.023 (0.944 to 1.109) per kg | 0.579 | Overall treatment effect remained significant; body weight was not independently associated |
| *Notes: Global treatment effects were evaluated by robust Wald χ² tests for median and log-linear models and penalized likelihood-ratio χ² tests for Firth models. CII was exploratory. A lower score or count is favorable; OR >1 indicates a higher probability of a favorable categorical outcome.* | | | | | | | |

Supplementary Figure S1. The TIC picture of clove oils

Supplementary Figure S2. Paired hematological profiles of dogs at D0 and D14.

(A) White blood cell count (WBC). (B) Red blood cell count (RBC). Each thin line connects the D0 and D14 measurements from the same dog. Open and filled circles denote individual D0 and D14 observations, respectively. Large circles and error bars denote the group mean and 95% confidence interval (n = 20 dogs per group). Group × time P values were obtained from Gaussian repeated-measures models with an exchangeable working correlation structure. The connecting lines represent paired observations at the two prespecified sampling time points and do not imply measurements on intervening days.

Supplementary Figure S3. Paired serum biochemical profiles of dogs at D0 and D14.

(A) Globulin concentration (GLO). (B) Albumin-to-globulin ratio (A/G). Each thin line connects the D0 and D14 measurements from the same dog. Open and filled circles denote individual D0 and D14 observations, respectively. Large circles and error bars denote the group mean and 95% confidence interval (n = 20 dogs per group). Group × time P values were obtained from Gaussian repeated-measures models with an exchangeable working correlation structure. The connecting lines represent paired observations at the two prespecified sampling time points and do not imply measurements on intervening days.

Groups: Control, untreated control group; Low-dose, clove oil at 0.05 mL/cm²; Middle-dose, clove oil at 0.10 mL/cm²; High-dose, clove oil at 0.20 mL/cm²; Amitraz, positive-control group treated with 0.05% amitraz at 0.10 mL/cm².
